# Impact of treatment of ADHD on intimate partner violence (ITAP), a study protocol

**DOI:** 10.1186/s12888-014-0336-2

**Published:** 2014-11-27

**Authors:** Nannet JL Buitelaar, Jocelyne A Posthumus, Agnes Scholing, Jan K Buitelaar

**Affiliations:** Psychiatrist at De Forensische Zorgspecialisten, Utrecht, The Netherlands; Radboud University Nijmegen, Nijmegen, The Netherlands; Associate Professor at University of Amsterdam, Amsterdam, The Netherlands; Clinical psychologist at Pro Persona Mental Health Institute, Nijmegen, The Netherlands; Department of Cognitive Neuroscience, Professor of Psychiatry and Child and Adolescent Psychiatry at Radboud University Medical Centre, Donders Institute for Brain, Cognition and Behaviour, Nijmegen, The Netherlands; Child and adolescent psychiatrist at Karakter, Nijmegen, the Netherlands

**Keywords:** Intimate partner violence, IPV, Domestic violence, Attention-deficit/hyperactivity disorder, ADHD, Treatment of intimate partner violence, Treatment of domestic violence

## Abstract

**Background:**

Attention-Deficit/Hyperactivity Disorder (ADHD) in adults is one of the predictive and treatable risk factors for delinquency, including intimate partner violence (IPV). Effective treatment of IPV needs to address personal dynamic risk factors, offender typology, and dynamics of the domestic violence. It is unknown whether treatment of ADHD symptoms contributes to a decrease in IPV. The ITAP study aims to investigate the relationship between treatment of ADHD symptoms and IPV in patients in forensic mental health care. Moreover, this study examines the role of comorbid psychopathology, subtype of the offender, and dynamics of the domestic violence.

**Methods/Design:**

The ITAP study is a longitudinal observational study. Participants are followed one year through various assessments: one before starting treatment (t0), and four during treatment (8, 16, 24 and 52 weeks after start of the treatment). All participants receive treatment for IPV, ADHD, and comorbid psychopathology, if present. The primary outcome measure is the change in severity of IPV; the primary predictive variable is the change in severity of ADHD symptoms. The secondary outcome measure is the observation of the therapist about change in the offender’s general violent behaviour, within and outside the partner relationship. Data are analysed in a multiple regression model with change in severity of IPV as the dependent variable and change in severity of ADHD symptoms as the primary predictor. Other predictive variables taken into account in the analyses are presence of comorbid psychopathology and personality disorder, subtype of the offender, and dynamics of the domestic violence. In addition, compliance with treatment and content of the treatment are documented.

**Discussion:**

Research on the treatment process of IPV offenders and victims is complicated by many factors. This observational design will not allow inferences about causality but may reveal clinically important factors that contribute to more effective treatment of IPV.

**Trial registration:**

The Netherlands National Trial Register (NTR), trial ID NTR3887.

## Background

The WHO defines domestic violence as “any behaviour within an intimate relationship that causes physical, sexual or psychological harm”. Studies about domestic violence reported a prevalence of nearly 40% in women who attended general practices [1,2]. In a national survey in the Netherlands, 9% of the general population reported severe victimisation of domestic violence in the past five years, mostly intimate partner violence (IPV) [3]. IPV seems to be the most common form of violence committed in the Netherlands. The violence reported included physical violence in 65% of the cases, sexual violence in 8% of the cases, and psychological violence in 28% of the cases. Sixty percent of the victims were female, whereas 83% of the offenders were male.

The consequences for victims of IPV and children who witness IPV are severe. In a WHO multi-country study on women’s health and domestic violence, women who experienced partner violence reported significantly more emotional distress, suicidal thoughts, and suicidal attempts than non-abused women [4]. In a US national sample of adolescents, the prevalence of parental violence witnessed was 9%. Having witnessed violence predicted posttraumatic stress disorder (Odds ratio (OR) 2.02, CI (95%) 1.22-3.32) and depressive disorder (OR 1.73, CI (95%) 1.11-2.71) [5].

Existing explanatory models about IPV can be divided into sociocultural, interpersonal, and intrapersonal theories. Sociocultural theories conceptualise IPV as a result of social structures and culturally determined views on the role of men and women. The best-known model within this theory is the Duluth model [6]. Interpersonal theories focus on different causes of IPV: the learning of aggressive behaviour by others (modelling and social learning theory), disturbed attachment patterns (attachment theory), and out-of-control conflicts. Relevant trends of these theories are the blended behaviour therapy [7], the emotionally focused therapy [8], and the solution focused therapy [9]. Intrapersonal theories emphasise the individual characteristics of the offender, such as hereditary factors (temperament), predisposition to anger and hostility, abuse of alcohol and drugs, and presence of mental disorders including personality disorders.

In the tradition of the intrapersonal theories, many authors proposed classification of offenders in categories. Holtzworth-Munroe and Stuart [10,11] suggested an offender typology based on 15 earlier typologies and on the presence of three dimensions: severity and frequency of marital violence, generality of violence (family-only versus extrafamilial violence), and psychopathology of the offender. They differentiated four subtypes of offenders, i.e. the overall violent/antisocial type, the dysphoric/borderline type, the slightly antisocial type, and the family-only/passive aggressive type. Several studies confirmed the validity of these types by means of cluster analysis [12–15]. Nonetheless, little is known about the inter-rater reliability and the clinical suitability of these offender types.

As a part of the interpersonal theory, Johnson [16,17] proposed four patterns of violence: intimate terrorism (IT), violent resistance (VR), mutual violent control (MVC), and common couple violence (CCV). These types are mostly based on the motivation for committing the violence. IT is defined as systematic violence in a general pattern of control by one partner over the other, VR as violence committed by victims against their abusive partners, MVC as violence occurring when both partners act in a violent manner, and battling for control and CCV as occasional outbursts of violence from both partners. A few studies supported the validity of the distinction between IT and CCV [18–20]. Several articles described the prevalence of these types being different in distinctive samples: in the general population the most common type was CCV; in women’s shelters the most common type was IT [17,20,21].

The high prevalence of IPV and the severe consequences for victims and children stress the need for effective treatment. However, two meta-analyses showed that the overall effectiveness of treatment of partner violence is small [22,23]. An explanation for this finding is the heterogeneity of the offenders, victims, and relationships as well as the fact that most treatment programmes were not adjusted to this heterogeneity. According to the Dutch multidisciplinary Guideline for Domestic Violence [24], the characteristics and psychopathology of the offender must be addressed to make treatment more effective.

Case control studies comparing IPV offenders to controls revealed some characteristics. Offenders were more likely to have witnessed parental DV in their families of origin and were more likely to have been abused as a child [25]; they had higher scores on narcissistic, antisocial, schizotypal, and borderline personality traits [26]; and they had more borderline symptoms, more mood problems, and less self-control [27]. Besides, several prospective studies discovered childhood antisocial behaviour, low verbal IQ and low verbal reasoning predictive for adult IPV [28,29].

Attention-Deficit/Hyperactivity Disorder (ADHD) is one of the dynamic risk factors for delinquency. Several studies have shown that impulsivity and hyperactivity in childhood specifically predict future delinquency, to a large part, but not only, through the association with conduct disorder (which is often followed by antisocial personality disorder (ASPD) in adults) [30–34]. In particular, ADHD is associated with impulsive or reactive violent aggression [35]. Some studies have found an association between childhood and/or adult ADHD and adult IPV, sometimes mediated by ASPD [36–40]. Hyperactivity and impulsivity, as core symptoms of ADHD, were considered to elevate the risk of aggression, overall and within intimate relations [36,40,41]. Inattention problems in itself were found to increase the risk for adult IPV [36]. This can be explained by the fact that inattention problems may lead to problems with listening to the other and perceiving complex social situations, which may increase the risk of interpersonal conflicts. In persons with poor conflict-handling skills, both problems may increase the risk of aggression. Besides, ADHD often occurs together with mood swings, problematic self-regulation and poor emotion regulation [42]. Self-regulation and emotion regulation are usually seen as salient qualities to handle conflicts in an intimate relationship, and consequently, important to prevent IPV [43]. In addition, the high comorbidity of ADHD with substance use disorder and antisocial personality disorder further raises the risk for delinquency and IPV [44,45].

Prevalence of ADHD in children is 5%, and 3% to 4% in adolescents and adults [46–49]. Prevalence of ADHD in forensic mental health care is not known exactly but might be high, given increased prevalence rates in prisoners: 38% in a Dutch study of detainees [50] and 40% in a Swedish study of inmates [51]. A recently published meta-analysis showed an ADHD prevalence rate of 26.2% in adult prison populations [52]. Research in an outpatient clinic for forensic mental health care in the Netherlands demonstrated that an ADHD diagnosis in male adults had been missed very often earlier in life [53]. Especially prior contacts with the law predicted a missed diagnosis in the past. The authors hypothesised that delinquency may mask ADHD and may lead to overemphasis on all sorts of behaviour problems, and underestimation of ADHD.

ADHD symptoms can be treated effectively in adults. The efficacy of stimulants in reducing ADHD symptoms for adults is well documented in several meta-analyses [54]. Data from a large Swedish sample of adults with ADHD recently showed that, among patients with ADHD, crime rates were lower during periods when they were on ADHD medication [55]. In addition to drug treatment, psychological and psychosocial treatments are considered to be effective interventions for adults with ADHD facing the demands and responsibilities of adult life [56]. However, no data are known about the effect of ADHD treatment on rates and severity of IPV.

The ITAP study aims to investigate the impact of ADHD and its treatment within a comprehensive treatment approach of IPV. The rationale for this study is the hypothesis that successful treatment of ADHD symptoms, such as inattention, hyperactivity, impulsivity, and emotional instability, will decrease ADHD symptoms and lower the risk for and severity of IPV. In addition, the ITAP study attempts to reveal clinically important factors that contribute to a more effective treatment of IPV.

### Primary and secondary objective of the ITAP study

The primary objective of the ITAP study is to examine the correlation between change in ADHD symptoms and change in IPV during a 16-week treatment in forensic mental health care for offenders of IPV who also meet criteria of adult ADHD diagnosis.

The secondary objective of the ITAP study is to examine the correlation between change in ADHD symptoms and change in indoor and outdoor aggression during a 16-week treatment in forensic mental health care for offenders of IPV who also meet criteria of ADHD.

In addition, the ITAP study attempts to analyse the role of potential other predictors of IPV on treatment outcome, measured at t0, such as: a) severity of ADHD symptoms, b) severity of borderline personality disorder problems, c) severity of antisocial personality disorder problems, d) presence of mood disorder, e) presence of anxiety disorder, f) presence of substance use disorder, g) subtype of the offender according to Holtzworth-Munroe and Stuart [57], h) dynamics of the domestic violence according to Johnson [17], and i) compliance with the treatment.

## Methods

### Study population

Participants are recruited from all patients (men and women) referred to De Waag Utrecht and De Waag Amersfoort in the period from October 2012 to December 2015. De Waag is a forensic outpatient clinic in the Netherlands (locations in Amsterdam, Rotterdam, The Hague, Leiden, Haarlem, Utrecht, Amersfoort, and Almere). Patients are referred to De Waag by court, the probation service, or the primary health care service because of having committed an offence. All patients receive information about the ITAP study at home before intake. The professional who conducts the intake interview asks patients to participate and to sign informed consent.

### Design

Given existing evidence of effective treatment of ADHD in adults, it was considered ethically incorrect to conduct a randomised controlled trial with a condition in which ADHD is not treated. Therefore, we chose a longitudinal observational design with the focus on predictors for successful treatment of IPV. All patients with recent IPV are asked to participate in the study upon entry at De Waag. After informed consent, patients are interviewed by a trained clinician to diagnose possible adult ADHD or other axis I pathology and axis II pathology. Subsequently, those who are diagnosed with adult ADHD will be included in the study. Next, participants are followed for a period of one year by way of repeated assessments: one before (t0) and four during treatment (at 8, 16, 24 and 52 weeks after start of treatment, respectively). The assessments measure the severity of ADHD symptoms, IPV and indoor and outdoor aggression. All participants receive treatment for IPV, ADHD, and other psychopathology, if present. In addition, compliance with treatment and content of the treatment is monitored.

Treatment of all participants is based on the Risk Need Responsivity (RNR) model [58] and matched care. The treatment for IPV is based on the Dutch Guidelines for Familial/Domestic Violence in children and adults [24]. The Guidelines recommend partner-relation and/or family therapy, treatment of individual psychopathology, and training in communication skills. In the ITAP study, treatment of IPV consists of building a safety plan with the offender, partner, and children, if any, according to the Signs of Safety method of Turnell [59]. Furthermore, communication skills are trained according to the method of Murphy and Eckhardt [60]. The skills training comprises putting in time-out and improving communication and coping skills that are specifically relevant for intimate partner relations. The training is provided to individuals, couples, or groups. Treatment for ADHD is based on the European consensus statement on diagnosis and treatment of adult ADHD [56]. In the ITAP study, treatment of ADHD consists of psycho-education, medication, and skills training.

Other psychopathology is treated according to evidence-based interventions and guidelines. A psychiatrist provides the drug treatment; a family therapist partner relation therapy, and a psychologist psycho–education and skills training.

### Ethics statement

The ITAP study is performed in accordance with the ethical principles laid down in the 1964 Declaration of Helsinki and subsequent amendments. The study is granted an exemption from requiring ethics approval by the medical ethical committee (Commissie Mensgebonden Onderzoek –CMO- Region Arnhem-Nijmegen).

Registration number 2012/222; NL number 41031.091.12.

### Trial registration

The ITAP study is registered at the Netherlands National Trial Register (NTR), trial ID NTR3887.

### Instruments

#### DIVA 2.0

The structured diagnostic interview for ADHD in adults 2.0 (DIVA 2.0) was first developed in Dutch by J.J.S. Kooij, MD PhD, psychiatrist, and M.H. Francken, MSc, psychologist in 2007 [61]. Since October 2010, a slightly adjusted version with an improved introduction of the DIVA has been available, DIVA 2.0. The DIVA is freely available via www.divacenter.eu. The interview allows a structured assessment of the DSM IV criteria for ADHD in adulthood and (retrospectively) in childhood. In addition, the severity of dysfunction due to ADHD symptoms is assessed in five areas: work and education, relationship and family life, social contacts, leisure time, and self-confidence and self-image. Clinical experience with ADHD diagnostics is a prerequisite for adequate use of the interview. Each criterion is scored on the basis of the information obtained from the patient (if possible, also the family) and the clinical judgement of the clinician. The DIVA 2.0 yields both a categorical outcome (ADHD is present/absent) and a dimensional outcome (the number of criteria and areas of dysfunction scored). In this study, a patient is considered to have an ADHD diagnosis when at least 6/9 criteria for attention deficit and/or hyperactivity-impulsivity in childhood and at least 5/9 criteria of attention deficit and/or hyperactivity-impulsivity in adulthood, and at least 2 areas of dysfunction in childhood and adulthood are present (according to DSM-5 [62]). No results are available yet on the psychometric properties of the DIVA 2.0. However, the interview is used in many countries, it was used to estimate the prevalence of ADHD in older adults in the Netherlands [63], and – as it is a structured interview based on DSM IV TR criteria – it is a widely used instrument for diagnosing ADHD in adults.

#### PDQ R

The Personality Disorder Questionnaire Revised (PDQ R) is a screening instrument for DSM-IV-R personality disorders. Sensitivity is high, specificity is low [64]. The PDQ R yields dimensional scores for the various personality disorders of DSM-IV-R. In this study, the PDQ R is used as a screening tool prior to the SCID2 interview. The PDQ R cut-off point per personality disorder is the same as the required number of criteria according DSM-IV-R per personality disorder.

#### SCID2

The Structured Clinical Interview for DSM IV Axis II diagnoses (SCID2) [65] is a generally accepted and widely used semi-structured interview for assessing Axis II diagnoses according to DSM IV. The score on the SCID2 is both categorical (personality disorder is present/absent) and dimensional (the number of criteria scored per personality disorder). Kappa for inter-rater reliability ranged from .48 - .98 for categorical diagnoses; intra-class correlation coefficients ranged from .90 - .98 for dimensional assessments; internal consistency coefficients ranged from .71- .94 [66]. In the ITAP study, only the sections on the personality disorders with PDQ R scores above cut-off points will be administered.

#### MINI plus

The Mini International Neuropsychiatric Interview Plus (MINI plus) is a structured interview for assessing diagnoses on Axis I according to DSM IV. Several studies showed adequate psychometric properties of the MINI plus [67,68].

#### ADHD RS for adults, Dutch version

The ADHD DSM-IV Rating Scale (ADHD RS) [69] is a widely used self-report measure of efficacy in clinical trials of ADHD treatment in children and adolescents [70,71]. An adult version was developed and validated at New York University and used in adult populations with the purpose of measuring treatment efficacy [72,73]. Goodman et al. [74] found that an improvement of 8–10 points (25%) on the ADHD RS corresponded clinically with a 1-point improvement on the Clinical Global Impression of Severity (CGI-S), and that an improvement of 16–20 points (50%) on the ADHD RS corresponded with a 2-point improvement on the CGI-S.

This study uses the Dutch version of the ADHD RS for adults. This version contains 23 items. The Dutch version has good internal consistency (alpha 0.81 – 0.85 for the patient version and 0.83 – 0.88 for the partner version) [75] and is commonly used in Dutch clinical trials to evaluate treatment [76].

#### CTS2

The Conflict Tactics Scales (CTS) [77] is the most widely used research method for identifying IPV. The CTS starts from the premise that conflict is an inevitable aspect of all human relationships, but that the use of coercion (including force and violence) as a conflict-resolution tactic is harmful. The Revised Conflict Tactic Scales of the CTS (CTS2) [78] is a self-report questionnaire consisting of 39 items that ask about aspects of behaviour of both the respondent and the partner. The score reflects the frequency of the behaviour during a certain period. In this study an eight-week time frame was used to make it possible to measure any differences through therapy. A similar approach was followed by Kraanen et al. [79]. The CTS2 consists of five subscales: 1. Physical assault (score between 0 and 300); 2. Psychological aggression (score between 0 and 200); 3. Injury (score between 0 and 175); 4. Sexual coercion (score between 0 and 175), and 5. Negotiation (score between 0 and 150). A limitation of the CTS2 is that the scales count acts of violence but do not provide information about the context in which the violence occurs. The CTS2 also does not measure manipulation involving children, isolation, or intimidation and fails to detect ongoing systematic patterns of abuse. Despite these limitations, the CTS2 was chosen as a primary outcome measure for IPV since there is no better outcome measure known in literature. Mean scores on the Physical Assault subscale in a sample of 183 male patients with alcohol substance abuse and partner violence were 9.4 (SD 15.8, median 4; range 1–129) and mean scores on the Injury subscale were 6.8 (SD 8.8, median 4, range 1–54) [80]. In the ITAP study both participants and partners will fill in the CTS2. Scores are analysed separately and consistency between the two partners is calculated.

#### MOAS

The Modified Overt Aggression Scale (MOAS) [81]) is a measure that describes different forms of aggression in psychiatric patients. The MOAS consists of four categories of aggressive behaviour: verbal aggression, physical violence against objects, physical aggression against oneself, and physical violence against other people. Each category is scored on a four-point scale; the highest score is the most severe one. The MOAS is scaled by a clinician on the basis of own clinical observation, information from the patient, and information from other people. It’s psychometric properties were studied in a psychiatric ward in New York; the observation scale proved to be reliable and valid [81].

For the purpose of this study, we adapted the MOAS to the subject of partner violence to have a second outcome measurement. We left the aggression against oneself out and we asked about behaviour of indoor and outdoor aggression (verbal, physical and against objects). Because this version of the MOAS has not been validated for this sample, we ask both the patient and the therapist to fill in the questionnaire separately in order to calculate consistency between self-ratings and therapist-ratings.

### Subtype of the offender

Holtzworth-Munroe and Stuart [10,57] differentiated four subtypes of offenders, i.e. the overall violent/antisocial type, the dysphoric/borderline type, the slightly antisocial type, and the family-only/passive aggressive type. Subtype of the offender is scored by the therapist after the first three sessions on the basis of all available information. All therapists are trained to score the subtype of the offender. In order to increase reliability, the scoring is regularly discussed in the multidisciplinary team. In addition, part of the sample is scored simultaneously by trained students, in order to calculate inter-observer reliability of this categorisation.

### Dynamics of the domestic violence

Johnson [16], (2004) proposed four patterns of violence: intimate terrorism, violent resistance, mutual violent control, and common couple violence. The therapist scores the dynamics of the domestic violence after the first three sessions on the basis of all available information. All therapists are trained to score the dynamics of the domestic violence. In order to increase the reliability, the scoring is regularly discussed in the multidisciplinary team. In addition, a part of the sample is scored simultaneously by trained students, in order to calculate inter-observer reliability of this categorisation.

### Procedure of the ITAP

At t0 all participants fill in ADHD RS, CTS2 client version, MOAS client version, and PDQ R. The patient’s partner is asked to fill in the CTS2 partner version. Subsequently, a trained clinician administers three clinical interviews, i.e. SCID2, MINI plus and DIVA 2.0 in order to assess proper diagnoses of Axis I, Axis II, and adult ADHD. In addition, the treating therapist fills in the MOAS therapist version and assesses the subtype of the offender according to Holtzworth-Munroe and Stuart and the dynamics of the domestic violence according to Johnson. Only participants with severe IPV and ADHD diagnosis assessed by DIVA 2.0 (see inclusion criteria) are included in the ITAP study.

At t1 (8 weeks after start of treatment), t2 (16 weeks after start of treatment), t3 (24 weeks after start of treatment), and t4 (52 weeks after start of treatment) all participants fill in the CTS2 client version, the ADHD RS, and the MOAS client version. Partners fill in the CTS2 partner version. Therapists fill in the MOAS therapist version and assess compliance with treatment.

### Inclusion criteria of the ITAP study

*There are six inclusion criteria for the ITAP study:*

1. The participant must be 18 years of age or older.
2. The participant must be decisionally competent and willing to give informed consent.
3. The participant must have sufficient command of the Dutch language to fill in questionnaires.
4. The participant must have completed the self-report questionnaire on IPV in the period of 8 weeks prior to intake.
5. The severity of the IPV is at least moderate, operationalised by a score of at least 1 on the physical assault subscale of the Conflict Tactics Scale (CTS2), or a score of at least 1 on the injury from assault subscale of the CTS2, or a score of at least 3 on the psychological aggression subscale of the CTS2, or a score of at least 2 on the indoor aggression part of the MOAS.
6. Presence of ADHD diagnosis according to the DSM-5 as established on the basis of a clinical assessment: the DIVA.

### Exclusion criteria of the ITAP study

Patients with acute severe mental symptoms that require immediate crisis interventions will be excluded. This is assessed at the beginning by the professional who conducts the intake interview with the patient and during treatment by the treating therapist.

### Variables

The primary study outcome is the change in severity of IPV between t0 (before starting treatment) and t2 (after 16 weeks of treatment). The change in IPV is calculated by the difference in scores on the CTS2 client version between t0 and t2.

The secondary study outcome is the observation of the therapist about any change in indoor and outdoor aggression, determined by the difference in scores on the MOAS therapist version between t0 and t2.

The primary predictive variable is the change in ADHD symptoms between t0 and t2, determined by the difference in scores on the ADHD RS between t0 and t2. Compliance with treatment, as determined by the number of treatment sessions received at t2, is a covariate predictor.

Other covariate predictors, scaled at t0 are:

- Severity of ADHD: assessed by the DIVA 2.0; dimensional score;
- Severity of borderline personality disorder problems, assessed by the SCID2; dimensional score;
- Severity of antisocial personality disorder problems, assessed by the SCID2; dimensional score;
- Presence of mood disorder, assessed by the MINI plus; categorical score;
- Presence of anxiety disorder, assessed by the MINI plus; categorical score;
- Presence of substance use disorder, assessed by the MINI plus; categorical score;
- Subtype of the offender by Holtzworth-Munroe and Stuart, assessed by structured clinical assessment by the therapist; categorical score;
- Dynamics of the domestic violence by Johnson, assessed by structured clinical judgment by the therapist; categorical score.

### Sample size

Data will be analysed in a multiple regression analysis with change in IPV as dependent variable and change in ADHD symptoms as primary predictor. Taking alpha = 0.05, two-sided, a sample size of 131 participants at t2 has 80% statistical power to detect a standardised regression-coefficient of 0.25. With this sample size, a beta of 0.25 corresponds to an effect size of 0.5, which is considered a clinically relevant effect. A maximum of 10 predictors is included in this analysis. Given the target sample, a drop-out rate of 25% has to be taken into account. This means that the desired sample size at t0 is 164 participants.

### Data analysis

First of all, data will be analysed with SPSS in a multiple regression analysis with change in IPV at t2 as dependent variable and change in ADHD symptoms at t2 as primary predictor. Covariate predictors (compliance with treatment, severity of ADHD at t0, severity of borderline personality disorder problems, severity of antisocial personality disorder problems, presence of mood disorder at t0, presence of anxiety disorder at t0, presence of substance use disorder at t0, subtype of the offender by Holtzworth-Munroe and Stuart, and dynamics of the domestic violence by Johnson) will be included in the analysis. The primary analysis is with completers. Additionally, an intention to treat analysis will be carried out using the last-observation-carried-forward method.

Later on, an additional and exploratory analysis will be carried out in which all time points are examined by using a multilevel-mixed model approach.

## Discussion

The high prevalence of IPV and the severe consequences for victims and children stress the importance of focusing on adequate therapy. However, little is known about effective treatment of IPV. The primary objective of the ITAP study is to examine the role of ADHD and its treatment within a comprehensive treatment approach of IPV. The ITAP study attempts to demonstrate the importance of detecting and treating ADHD as one of the dynamic risk factors for IPV. Our primary hypothesis is that ADHD treatment response will lower the risk for IPV. If this hypothesis is confirmed, this insight may contribute to the practice of diagnostics and treatment of IPV offenders. Additionally, the ITAP study examines the presence of comorbid psychopathology and includes this information in the analysis. Hence, results of the ITAP study are also expected to provide more insight into the contribution of comorbid psychopathology and its interaction with ADHD to the extent and severity of IPV. Furthermore, the ITAP study is expected to reveal more information about the importance of determining offender types, dynamics of the relationship and, finally, how this knowledge will influence the treatment of IPV. Although the prevalence of these classifications in IPV has been studied thoroughly, the benefits of these classifications within the treatment of IPV have never been examined. In most studies, offender types were determined by cluster analysis and not by clinical assessment. Little is known about the reliability of these parameters, when classified by clinicians for practical use. The inter-rater reliability is presumably problematic; therefore, data from the ITAP study can also be used for establishing this reliability.

Exploring the relationship between ADHD and IPV in a longitudinal design is challenging and research in this field will have to address several practical and methodological obstacles. Firstly, as mentioned above, a randomised controlled trial would provide stronger evidence for causality; however a design in which one group of ADHD patients is not treated is considered unethical. This observational design will not allow inferences about causality, but we hope to detect a strong association between severity of ADHD symptoms and that of IPV. Secondly, relationships of couples with IPV are usually very unstable. This may lead to problems with follow-up assessments, since change in IPV can only be measured if couples have at least regular contact. For this reason we extended the CTS2- and MOAS follow up questionnaires with questions about the actual situation: still living together, not living together anymore but frequently having contact, separated but still having contact, and contact with children, if any. Thirdly, the drop-out rate in forensic psychiatry is generally high and even higher in ADHD patients. Therefore, in estimating the sample size, a drop-out rate of 25% was taken into account. Fourthly, forensic patients tend to have little insight in their behaviour and problems or are inclined to trivialise it. This may result in lower scores on t0 self-reports and make it difficult to detect change. In addition, one of the intended effects of therapy is to improve self-reflection and self-understanding. Treatment with ADHD medication, for instance, often provides more insight into problematic behaviour. There is a slight possibility that this mechanism results in higher scores on follow up self-reports, such as ADHD RS, CTS2, and MOAS client version. In that case, a feasible decline of symptoms or improvement in behaviour is not properly reflected by the scores on the self-reports. Scores on the MOAS therapist version and the CTS2 partner version can meet these objections for the part of the change in IPV; in the case of a change in ADHD symptoms, it is harder to overcome these objections.

In spite of this, the ITAP study will provide more insight into the complex matter of treatment of IPV in clinical practice and may reveal clinically important factors that contribute to more effective treatment of IPV.
